# Laparotomy for Fibro-Cystoma: The Case of Natividad Krishbaum

**Published:** 1890-09

**Authors:** D. Berrey

**Affiliations:** Secretary, San Antonio, Texas


For Daniel's Texas Medical Journal.

### LAPAROTOMY FOR FIBRO-CYSTOMA.

### History of the Case of Natividad Krishbaum, Operated January 14, 1890.

Read at the Meeting of the West Texas District Medical Association by D. Berrey, M. D., Secretary, San Antonio, Texas.

ON TUESDAY, June 10th, I was called to see Mrs. Natividad Krishbaum, a Mexican woman, native of Durango, Mexico. On the day previous the patient had been brought to the city from Cotula, Texas, for treatment. I obtained from herself and husband the following history. She was 45 years of age, the mother of one child, and fifteen years before had had an abortion in the early months of pregnancy. Her health had been perfect up to one year ago, when she noticed an enlargement of the abdomen, which rapidly increased in size until the present time when her proportions are simply enormous. The menstrual function she claimed was regular, and her general health good. She only complaining of a slight dyspnœa and œdema of the lower extremities, the two last symptoms being very recent, having only been noticed a day or two before my seeing her. On examination the abdomen was found to be enormously distended, and rather irregular in outline, the enlargement seeming to be most prominent between the umbilicus and ensiform cartilage; on the right side could be felt a hard mass which could be fairly well outlined. On palpation fluctuation could be made out ove the entire abdomen. Vaginal examination revealed little save that the uterus was anteverted to such an extent from pressure from above that it was found to be impossible to introduce a sound, and protruding through the os was a small mucous polypus. The uterus moveable, and the os and cervix seemed natural to the touch. Diagnosis, fibro-cystoma of the right ovary with a possible cystoma of the left. I explained to the family the gravity of the case, and that the only possible hope for the patient was a laparotomy, and advised against delay. I may state here that before my seeing her she had been treated by an irregular physician who had cupped her, and was using lotions over the abdomen. The family desiring further counsel, Dr. George Cupples was requested to see the patient. The diagnosis was confirmed by Dr. C., and a laparotomy decided upon as the only means of saving her life, as soon as the necessary preparations could be made. The patient was eager for the operation, and her husband accepted it as the only alternative.

Dr. Cupples and myself took the following dimensions of the patient: Height five feet; from the ensiform cartilage to the umbilicus sixteen inches, from the umbilicus to the pubic arch nine and one-half inches; from the right anterior superior spinous process of the ilium to the left thirty-two inches, girth at the most prominent point fifty-four inches. On the day of the operation, three days after these dimensions were taken, the girth had increased two inches, making on the day of operation a difference of only four inches between her height and diameter around the body at the most prominent part of the mass. For preparatory treatment the patient was ordered liquid diet, a saline cathartic was to be given once or twice daily, and the entire body to be scrubbed once daily with warm water, soap, and a flesh brush.

June 14, four days after I first saw her was the date fixed for the operation. At noon of June 14, Dr. Cupples operated, present and assisting Drs. Berrey, Kingsley, Watts, Garza and Chew, the latter administering chloroform, a four inch incision through a thick fatty layer, revealed, on opening the peritoneum the glistening, silvery wall, of an enormous cyst, which was evacuated by means of a Spencer Wells trocar, nineteen quarts of colloid matter, varying in consistency from nearly pellucid serum, to pultaceous masses of different color, being furnished by this cyst alone. At this juncture there was some failure of the pulse due to removal of the great pressure, but this had been anticipated, and was remedied by a hypodemic injection of ether. Numerous other cysts were emptied, each presenting the color and density determined by its age. It was now possible to ascertain the relations of the immense tumor to the surrounding viscera, to all of which there existed adhesions of varying degrees of density, the whole omentum being closely adherent. The upper abdomen was enormously distended, and the adhesions firm and numerous. After having ligated and divided the almost innumerable attachments of the neoplasm, the pedicle was secured by transfixion and double ligation and divided at a distance of one inch from the uterus. In the depth of the pelvis considerable hemorrhage took place but it was finally controlled by ligature. The ragged remains of the omentum were ligated anew and cut away. The abdominal cavity was thoroughly sluiced with water, a glass drainage tube inserted, and the incision which had been extended to nine inches was closed by deep silver wire and superficial silk sutures. During this long and tedious operation, lasting two hours, two hypodermic injections of ether and three of whisky were given. There being absolutely no history of pain or discomfort in the abdomen at any time, the operator and his colleagues were much disappointed and surprised at the number and extent of the adhesions.

The patient was placed in bed, bottles of hot water put around her, and in a short time she rallied from the chloroform, without any appearance of shock. Perfect rest was enjoined, and iced milk in small quantities was ordered at stated intervals.

Nine p. m., found the patient resting comfortably, reaction was complete, and no evidence of a shock was visible. She had suffered some from chloroform nausea, but at my visit this had entirely passed off. Temperature normal, pulse 80.

June 15, 9 a. m. Found the patient comfortable. Had passed a good night, voided urine freely, complains of no pain, takes the iced milk with relish, and asks for solid food. Temperature normal.

At 5 p. m., Dr. Cupples and myself were hastily summoned to the patient, and found her with a temperature of 102 Fahr., abdomen considerably tympanitic. We partially removed the dressing, and found the drainage tube clear, probably an ounce of sero-sanguinous fluid having escaped through it. We removed the short glass drainage tube, and substituted a long rubber one, gave the patient a water and soap enema, ordered one dram of suph. of magnesia to be given by enema every hour, and left the woman feeling fairly easy. She rested well during the night, but on Monday morning the 16th she was taken with a sudden attack of vomiting and died before Dr. Cupples or myself could reach the house, forty-five hours after the operation.

Autopsy forty-eight hours after the operation, and three hours after death. The incision was firmly united except a small point at the umbilicus. The abdomen contained a considerable quanity of serum, untinged by blood, showing that complete hemostasis had been secured. Death was evidently due to what Mr. Bryant of St. Thomas Hospital, London, has happily termzd "shock of peritonitis," the patient having passed a quiet night, and reported herself better on awakening from sleep, not an hour before death. The sac, empty, weighing nearly 10 pounds, the whole contents by actual measurement, and estimated weight over eighty pounds. In addition to the mucous polypus occupying the os, the uterus presented several polypi, sub-peritoneal sub-mucous, and intra-mural.

